# 2,2′-Bipyridyl formation from 2-arylpyridines through bimetallic diyttrium intermediate†
†Electronic supplementary information (ESI) available: Experimental details for the synthesis and characterization of Y complexes, ^1^H NMR spectrum of the deuterium labelling experiment, and crystal data for **3e** (CCDC 1409167), **4a** (CCDC 1048580), **4b** (CCDC 1048581), **4c** (CCDC 1048582), **6a** (CCDC 1048583), **6c** (CCDC 1048584) and **6d** (CCDC 1048585). For ESI and crystallographic data in CIF or other electronic format see DOI: 10.1039/c5sc01599e



**DOI:** 10.1039/c5sc01599e

**Published:** 2015-07-17

**Authors:** Yu Shibata, Haruki Nagae, Shiki Sumiya, Raphaël Rochat, Hayato Tsurugi, Kazushi Mashima

**Affiliations:** a Department of Chemistry , Graduate School of Engineering Science , Osaka University , Toyonaka , Osaka 560-8531 , Japan . Email: mashima@chem.es.osaka-u.ac.jp ; Email: tsurugi@chem.es.osaka-u.ac.jp

## Abstract

An alkylyttrium complex supported by an *N*,*N*′-bis(2,6-diisopropylphenyl)ethylenediamido ligand, (ArNCH_2_CH_2_NAr)Y(CH_2_SiMe_3_)(THF)_2_ (**1**, Ar = 2,6-^*i*^Pr_2_C_6_H_3_), activated an *ortho*-phenyl C–H bond of 2-phenylpyridine (**2a**) to form a (2-pyridylphenyl)yttrium complex (**3a**) containing a five-membered metallacycle. Subsequently, a unique C(sp^2^)–C(sp^2^) coupling of 2-phenylpyridine proceeded through a bimetallic yttrium intermediate, derived from an intramolecular shift of the yttrium center to an *ortho*-position of the pyridine ring in **3a**, to yield a bimetallic yttrium complex (**4a**) bridged by two-electron reduced 6,6′-diphenyl-2,2′-bipyridyl. Aryl substituents at the *ortho*-position of the pyridine ring were key in order to destabilize the μ,κ^2^-(C,N)-pyridyldiyttrium intermediate prior to the C(sp^2^)–C(sp^2^) bond formation.

## Abstract 2

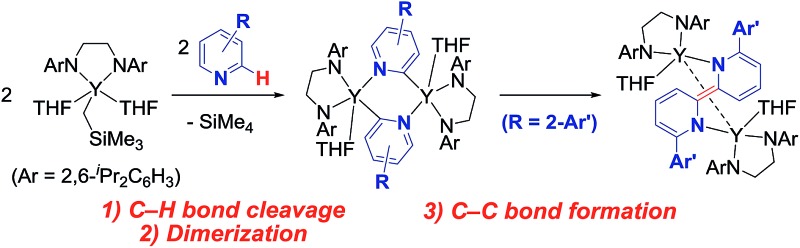
Formation of dianionic 2,2′-bipyridyl-bridged dinuclear yttrium complexes proceeded upon treatment of (ArNCH_2_CH_2_NAr)Y(CH_2_SiMe_3_)(THF)_2_ with 2-arylpyridine, in which mononuclear (2-pyridylphenyl)yttrium complexes were detected as key intermediates.

## Introduction

**Fig. 1:**
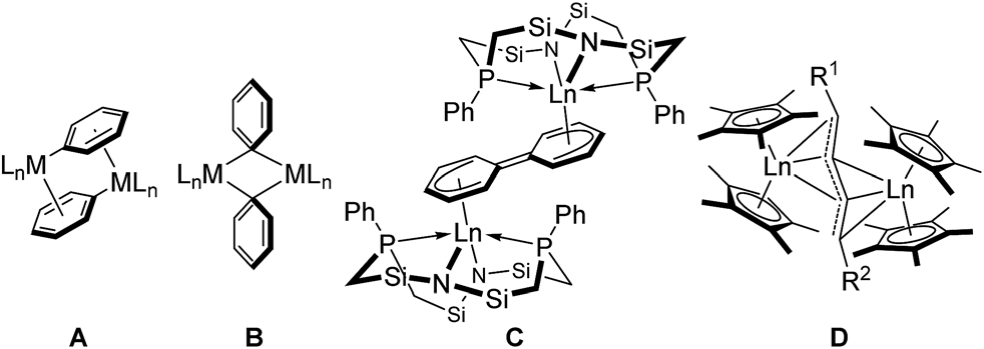
Aggregated monoarylated and monoalkynylated metal species. **A**: π-coordination-bridged bimetallic species. **B**: 3-centered-2-electron Ph-bridged bimetallic species. **C**: dianionic biphenyl-bridged bimetallic lanthanide complex. **D**: dianionic diyne-bridged bimetallic lanthanide complex.

Transition metal-catalyzed homo-coupling reactions of two arenes are an important category of C–C bond forming reactions to construct π-conjugated biaryl skeletons.^1,2^ For example, in the Ullmann coupling reaction, the activation of aryl C–X bonds of heteroarenes with a low-valent metal species, such as Cu powder or Ni(cod)_2_, produces the corresponding biaryl compounds. Although these reductive homo-coupling reactions are frequently used, the formation of salt waste is inevitable, and thus there is a demand for a more atom-economical method to synthesize biaryl skeletons. The most direct protocol for C(sp^2^)–C(sp^2^) bond formation is through C–H bond activation of aromatic compounds. To date, various transition metal complexes have been applied to such dehydrogenative biaryl coupling reactions.^3^ The most well-established mechanism for biaryl C(sp^2^)–C(sp^2^) bond formation mediated by a mononuclear species is reductive elimination of mononuclear di(aryl)metal species. However, some monoarylated metal species undergo biaryl C(sp^2^)–C(sp^2^) coupling reactions. In this reaction, two mechanisms are proposed to be involved, *i.e.* disproportionation to produce a di(aryl)metal and low-valent metal species,^4^ and an associative C–C bond formation mediated by two metal centers.^5,6^ In the associative mechanism, a bridged dimer species **A** is first formed through π-coordination of the aryl moiety to another metal center, followed by the formation of species **B**, which contains a 3-centered-2-electron bridging aryl moiety (Fig. 1). Subsequent C–C bond formation from species **B** produces the corresponding biaryl compound. A closely related reaction is the Glaser diyne coupling reaction of terminal alkynes using a Cu catalyst, the mechanism of which involves a stepwise process through π-coordination of the C≡C bond to a different metal center and a 3-centered-2-electron C(sp)-bridging dinuclear intermediate before the C–C bond-forming step.^7^ Synthesis of the C≡C π-coordination-bridged multimetallic species and mechanistic studies of the bimetallic aggregation-assisted C(sp)–C(sp) bond formation are feasible due to the strong coordinating ability of the alkyne moiety to the metal center; however, corresponding studies of arylmetal species and the mechanism of associative biaryl C(sp^2^)–C(sp^2^) bond formation have not been established due to the weaker π-aromatic coordination to the metal center compared with C≡C π-coordination.

**Scheme 1:**
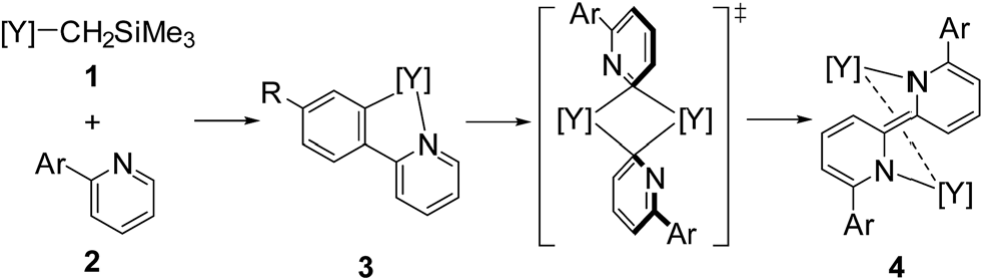
Pyridylyttrium-mediated 2,2′-bipyridyl formation. [Y] = (ArNCH_2_CH_2_NAr)Y.

Rare-earth metal complexes containing a mono(aryl)- or mono(alkynyl)metal moiety, generated by C–H bond activation of arenes and terminal alkynes using an alkylmetal species, also mediate C(sp^2^)–C(sp^2^) and C(sp)–C(sp) bond formation through the aggregation of two metal species. Because of the stability of the +3 oxidation state of the rare-earth metal center, C–C bond formation products, biaryls and diynes, have been trapped in their dianionic form to give bimetallic complexes such as **C** and **D** as reported by Fryzuk *et al.* (Fig. 1),^5,8,9^ even though access to low-valent rare-earth metal complexes has been reported by Evans *et al.* ^10^ In our studies on the C–H bond activation of heteroaromatic compounds by rare-earth metal and early transition metal complexes,^11^ we found that σ-bond metathesis and subsequent 2,2′-bipyridyl formation from 2-arylpyridines proceeded upon treatment of an alkyl complex of (ethylenediamido)yttrium (**1**) with 2-arylpyridine (**2**). During this transformation, the C–H bond adjacent to the nitrogen atom of the pyridine ring was selectively functionalized. Mononuclear (2-pyridylphenyl)yttrium complex **3** was detected and isolated as an intermediate in the formation of dianionic 2,2′-bipyridyl-bridged dinuclear yttrium complex **4** (Scheme 1). This is the first example of 2,2′-bipyridyl formation through bimetallic aggregation, even though dimerization of pyridine *via* C–H bond activation and insertion reactions has previously been reported by Teuben and Diaconescu, respectively.^12^ Catalytic 2,2′-bipyridyl formation *via* C–H bond activation has only been achieved using heterogeneous Pd/C and Ru cluster catalysts.^13^ In addition, steric and electronic tuning of the pyridine derivatives led to the isolation of dinuclear bis(μ,κ^2^-(C,N)-pyridyl)diyttrium, mononuclear κ^2^-(C,N)-pyridylyttrium, and 5-membered metallacycle complexes as possible intermediates in the C–C bond formation.

## Results and discussion

**Fig. 2:**
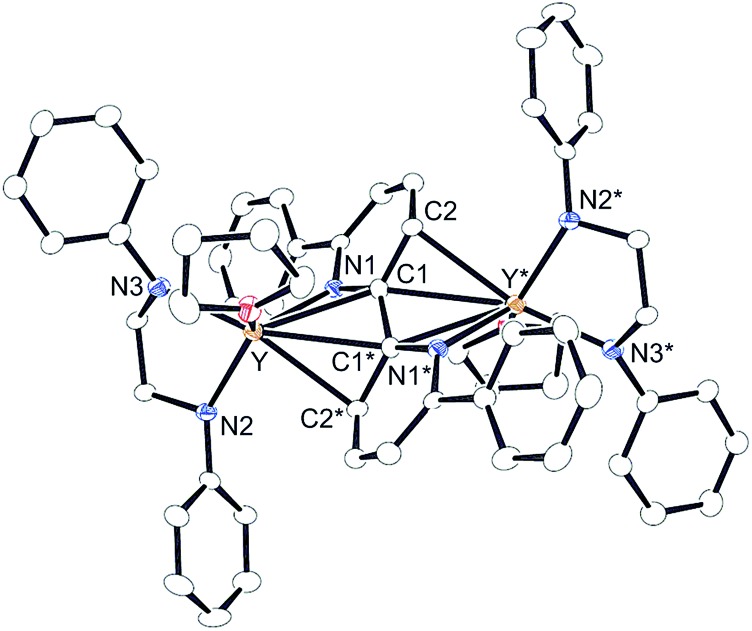
Molecular structure of complex **4a** with 30% thermal ellipsoids. All hydrogen atoms and isopropyl groups are omitted for clarity. Selected bond lengths (Å) and angles (°): Y1–N1, 2.344(5); Y1–C1, 2.746(5); Y1–C1*, 2.857(5); Y1–C2*, 2.661(5); N1–C1, 1.424(6); C1–C2, 1.476(7); C1–C1*, 1.396(10); Y1–N2, 2.194(4); Y1–N3, 2.192(5); N1–Y1–C2*, 70.68(16); N2–Y1–N3, 80.78(17). Dihedral angle between N1–Y1–C2* and N1–C1–C1*–C2* planes, 109.6.


We first treated an alkylyttrium complex (ArNCH_2_CH_2_NAr)Y(CH_2_SiMe_3_)(THF)_2_ (**1**, Ar = 2,6-^*i*^Pr_2_C_6_H_3_)^14^ with 1 equiv. of 2-phenylpyridine (**2a**) in benzene at room temperature. The color of the solution changed immediately from pale yellow to orange and then to dark green, and green-colored crystals of **4a** were precipitated (eqn (1)). The green crystals were sparingly soluble in aromatic and aliphatic solvents. The molecular structure of **4a** was determined by X-ray diffraction studies, and its ORTEP drawing is shown in Fig. 2. During the reaction, 6,6′-diphenyl-2,2′-bipyridyl was formed as a biaryl coupling product of 2-phenylpyridine. Two (ethylenediamido)yttrium moieties are bridged by the two-electron reduced 6,6′-diphenyl-2,2′-bipyridyl ligand. The nitrogen atoms in the 2,2′-bipyridyl moiety are located on opposite sides, and the 2,2′-bipyridyl ligand coordinates to the two yttrium atoms in a μ–η^4^:η^4^-coodination mode. The bond length of Y1–N1 (2.344 Å) is shorter than the typical yttrium–nitrogen dative bond (*ca.* 2.5 Å),^15^ but longer than the Y1–N2 and Y1–N3 bonds (*ca.* 2.19 Å). The Y1–C2* bond (2.661 Å) is much longer than the yttrium–carbon covalent bond (*ca.* 2.45 Å).^5b,15c^ The C1–C1* bond (1.396 Å) of the central 2,2′-bipyridyl moiety is similar in length to the analogous bond in two-electron reduced 2,2′-bipyridyl bound to two alkali metal centers with alternate planes (1.400 Å, rubidium).^16^

1

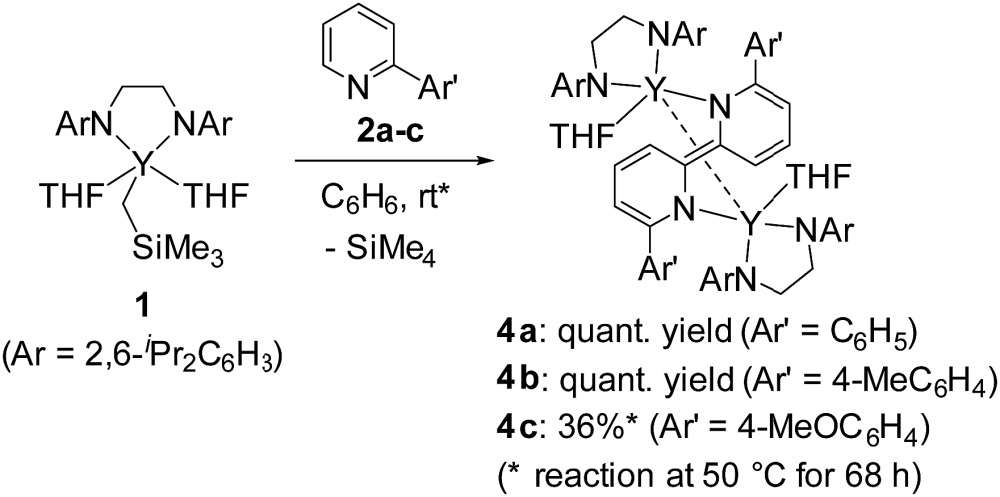



In addition to 2-phenylpyridine, 2-arylpyridines with methyl and methoxy groups at the *para*-position of the phenyl group were applicable to the 2,2′-bipyridyl formation. Complex **4b** was isolated in quantitative yield after treatment of **1** with 1 equiv. of 2-(4-methylphenyl)pyridine (**2b**) at room temperature for 48 h. When 2-(4-methoxyphenyl)pyridine (**2c**) was used as the substrate, heating the reaction mixture at 50 °C for 68 h led to the formation of green crystals of **4c** in 36% yield. The low isolated yield of **4c** was attributed to decomposition during the C–C bond forming process. Because complexes **4b** and **4c** had poor solubility in aliphatic and aromatic solvents and low stability in coordinating solvents, characterization of **4b** and **4c** was based only on X-ray diffraction studies and combustion analyses.^17^

**Scheme 2:**
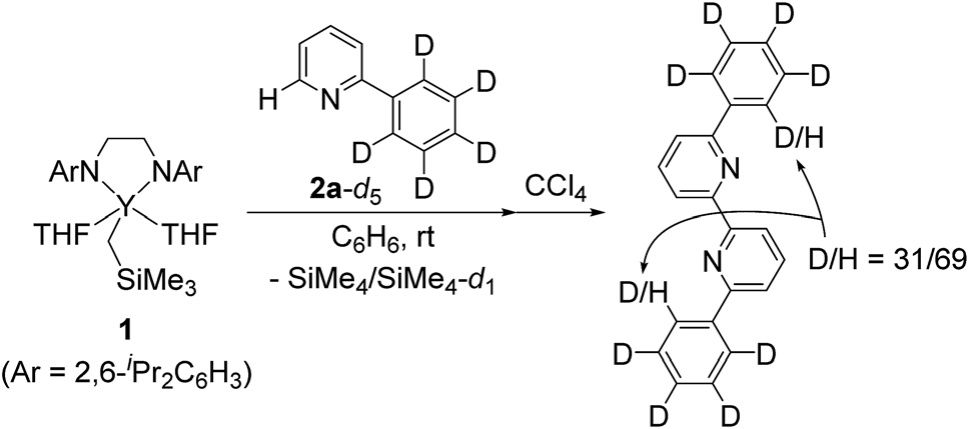
Deuterium labelling experiment.

We next conducted a deuterium labelling experiment. The addition of C_6_D_5_ derivative **2a**-*d*_5_ to complex **1** in C_6_H_6_ resulted in the formation of the same green crystals together with a mixture of SiMe_4_ and SiMe_4_-*d*_1_, the former indicating direct C–H bond activation at the *ortho*-position of the pyridine ring by the alkylyttrium moiety, and the latter indicating C–D bond activation of the *ortho*-C_6_D_5_ position by Y–CH_2_SiMe_3_ followed by an intramolecular shift of the yttrium atom to the *ortho*-position of the pyridyl before the C–C bond forming process (*vide infra*). These processes are consistent with subsequent oxidative quenching of the crystalline compound by CCl_4_ to give a mixture of *d*_8_-, *d*_9_-, and *d*_10_-6,6′-diphenyl-2,2′-bipyridyl, as evidenced by the intensity (69%-H) of the singlet signal corresponding to the *ortho*-position of the phenyl ring at *δ*_H_ 8.18 (Scheme 2).

**Scheme 3:**
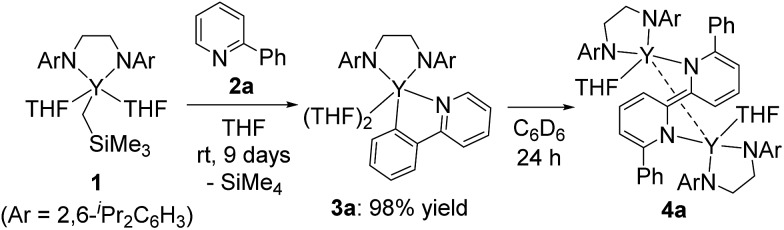
Stepwise metallacycle and C–C bond formation.

In addition, when **1** was reacted with **2a** at room temperature in THF for 9 days to allow complete C–H bond activation, 5-membered metallacyclic complex **3a** was isolated in 98% yield. Complex **3a** was stable and no further coupling reaction was detected in THF, but dissolution of **3a** in C_6_D_6_ afforded the bimetallic compound **4a** quantitatively. This clearly indicated that the 5-membered metallacyclic complex **3a** is a metastable species in benzene that could lead to a subsequent intramolecular shift of the yttrium center to the *ortho*-position of the pyridine ring, followed by C–C bond formation to afford **4a** (Scheme 3).

**Scheme 4:**
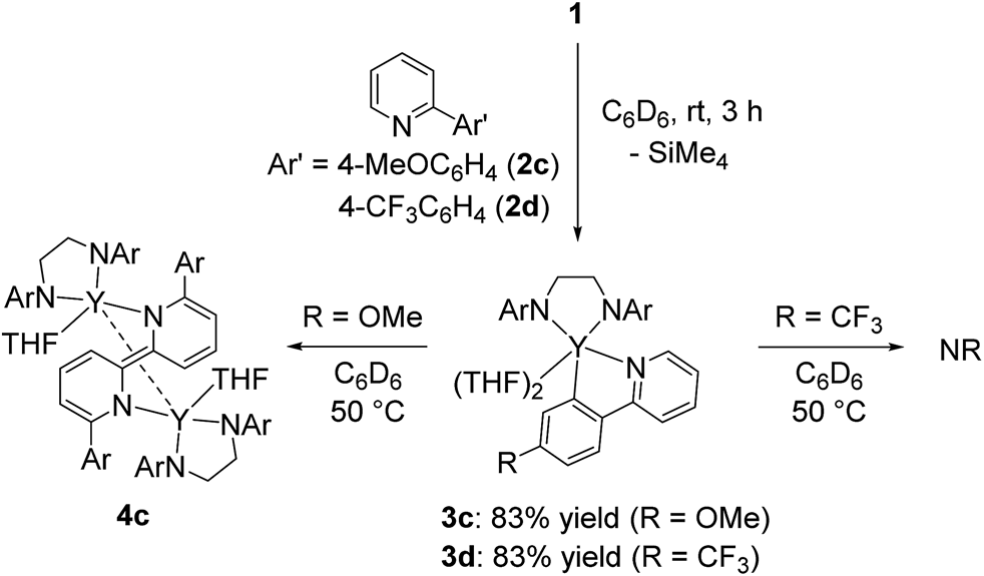
Effect of substituents on the aryl ring of 2-arylpyridine on the C–C bond formation step.

When 2-arylpyridines **2c** and **2d** were treated with **1** in C_6_D_6_ at room temperature, C–H bond activation of the aryl ring proceeded to form five-membered metallacyclic complexes **3c** and **3d** (Scheme 4). On heating the C_6_D_6_ solution of **3c** at 50 °C, green microcrystals were precipitated from the reaction mixture, as determined by eqn (1). In contrast, **3d** was stable in C_6_D_6_ at 50 °C. We thus presumed that the C–C bond formation was affected by electron-donating/-withdrawing substituents *meta* to the metallated carbon; THF coordination to yttrium for **3a** and **3b** was weaker than that for **3c** and **3d** in benzene, leading to easy dissociation of THF from yttrium for **3a** and **3b** and C–C bond formation to form **4a** and **4b** at room temperature.

In addition to the isolation of stable five-membered metallacyclic complex **3d**, the reaction of **1** with benzo[*h*]quinolone at room temperature gave **3e** in quantitative yield (eqn (2)). Complex **3e** was isolated as microcrystals suitable for X-ray diffraction studies. Although the quality of the crystallographic data from the X-ray diffraction studies was insufficient, we determined the overall structure of **3e**, in which a C–H bond of benzo[*h*]quinolone was activated to form a five-membered metallacycle as shown in the ESI (Fig. S1†). Complex **3e** was not converted to the C–C bond formation product analogous to **4a–c**, probably due to the low flexibility of the benzo[*h*]quinolone scaffold.
2

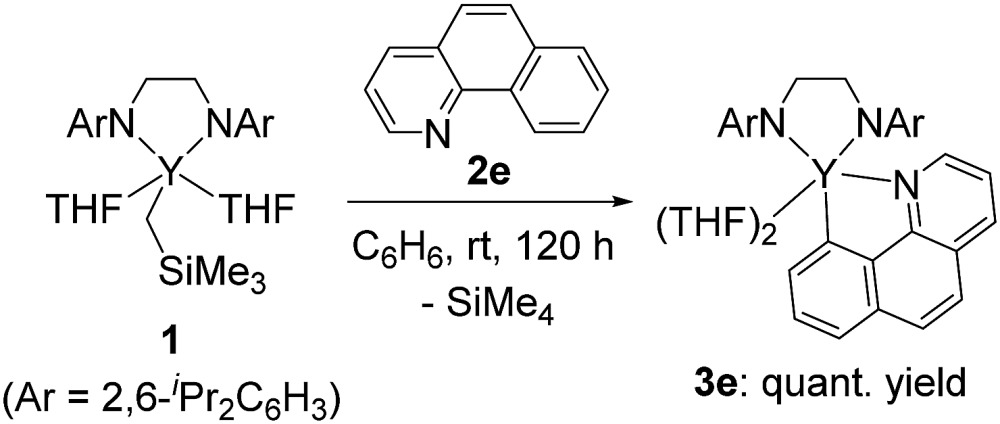




**Scheme 5:**
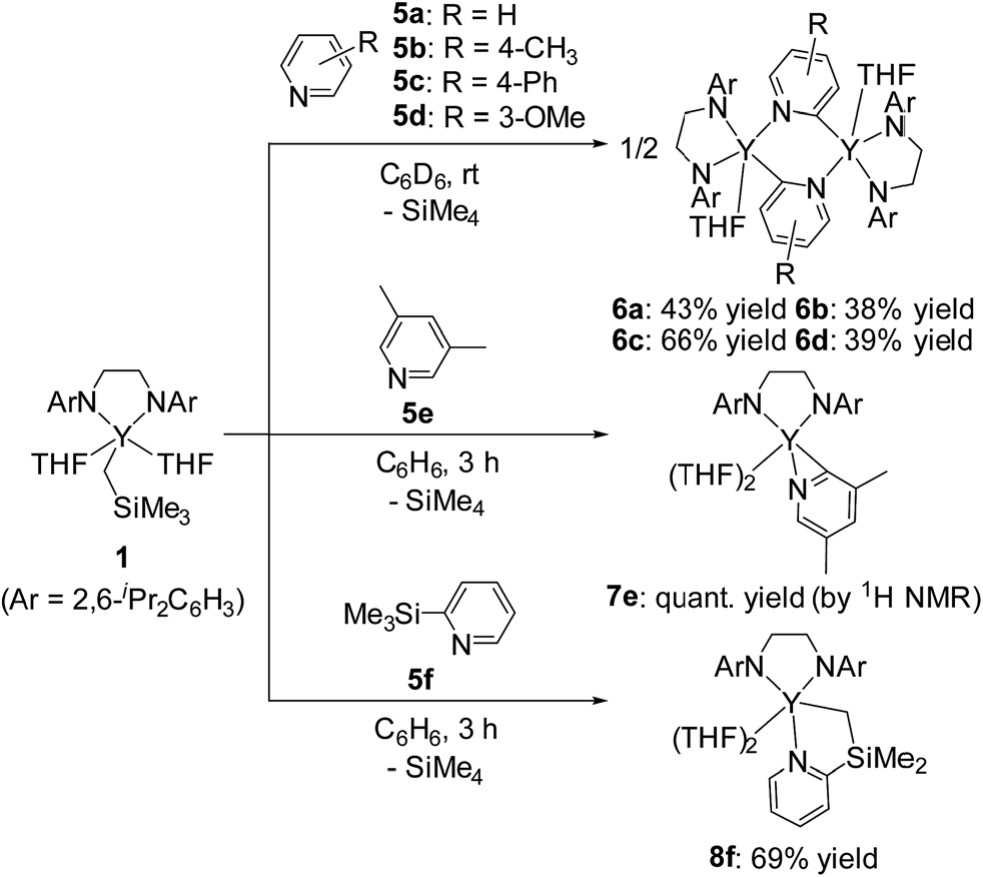
Reactions of alkylyttrium complex **1** with pyridine derivatives.

**Fig. 3:**
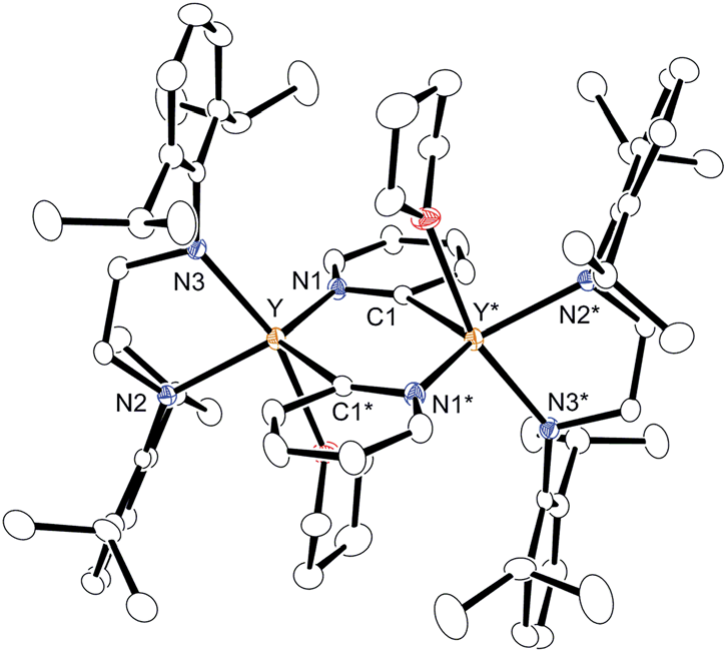
Molecular structure of complex **6a** with 30% thermal ellipsoids. All hydrogen atoms are omitted for clarity. Selected bond lengths (Å): Y1–N1, 2.328(4); Y1–C1, 2.681(5); Y1–C1*, 2.560(5); Y1–N2, 2.216(4); Y1–N3, 2.219(4); Y1–O1, 2.422(3).

In sharp contrast to the reaction of **1** with 2-arylpyridines **2a–2c**, treatment of **1** with 1 equiv. of pyridine (**5a**) or 4- or 3-substituted pyridines (**5b–d**) afforded bis(μ,κ^2^-(C,N)-pyridyl)diyttrium complexes **6a–d** as poorly soluble yellow microcrystals (Scheme 5). The ORTEP drawing of **6a** is shown in Fig. 3. The μ,κ^2^-(C,N)-pyridyl ligand is positioned at the bridging part of the bimetallic structure. The bond lengths of Y–N1 (2.328(4) Å) and Y*–C1 (2.560(5) Å) are longer than those found for mononuclear κ(C,N)-pyridylyttrium complexes.^17,18^ Two yttrium atoms, two bridging carbons, and the two nitrogen atoms of the bridging pyridines are located in the same plane. The ^1^H NMR spectrum of **6a** displays four resonances corresponding to the bridging pyridine ring at *δ*_H_ 9.18 (3-py), 8.81 (6-py), 7.46 (4-py), and 6.80 (5-py). A significant downfield shift of the resonance at the 3-py position might be due to the proximity of the C–H bond to the metal fragment. When 3,5-dimethylpyridine (**5e**) was used as the substrate, mononuclear yttrium complex **7e** was isolated in 97% yield. In the ^13^C NMR spectrum, a doublet signal was observed for the carbon atom attached to the yttrium center at *δ*_C_ 219.6 (^1^ *J*_Y–C_ = 35.2 Hz), which is in the typical range for mononuclear arylyttrium complexes.^18^ Even after heating solutions of complexes **6a–d** and **7e**, which contained a 2-pyridylyttrium moiety in the molecular structure, C–C coupling products were not detected in the reaction mixture; decomposition of the complexes was observed, and no single species was isolated from the reaction mixture. 2-Trimethylsilylpyridine (**5f**) was also reacted with yttrium complex **1** to form (dimethylpyridylsilyl)methylyttrium complex **8f** *via* C(sp^3^)–H bond activation. In this case, an intramolecular shift of the yttrium center to form 2-pyridylyttrium species or 6,6′-bis(trimethylsilyl)-2,2′-bipyridyl formation was not observed.

**Scheme 6:**
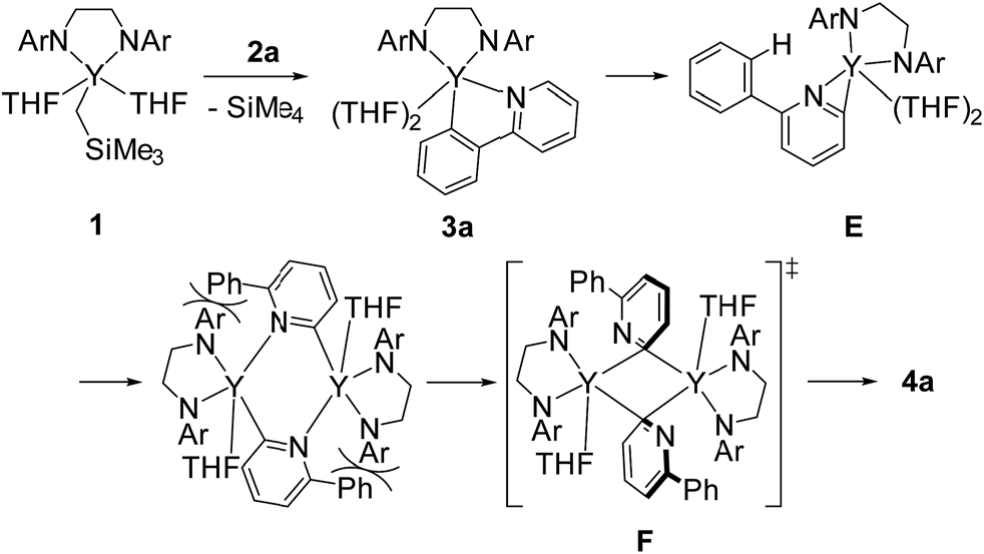
Plausible mechanism for the reductive dimerization of 2-phenylpyridine.

Based on our findings for the alkylyttrium-mediated C–H bond activation and C–C coupling reaction, we propose a mechanism for 2,2′-bipyridyl formation as shown in Scheme 6. First, alkylyttrium complex **1** cleaves a C–H bond at the *ortho*-position on the phenyl ring of **2a** to produce five-membered metallacycle complex **3a**. Complex **3a** is isomerized to three-membered metallacycle intermediate **E**. Initial formation of the five-membered metallacycle prior to formation of the three-membered metallacycle, as the major pathway, was confirmed by the deuterium labelling experiment as shown in Scheme 2, where mono-deuterated SiMe_4_-*d*_1_ was generated and one H atom was incorporated into the phenyl ring. Direct formation of the intermediate **E** from complex **1** was plausible as a minor pathway, and this was confirmed by the detection of SiMe_4_ in the deuterium labelling experiment. Isomerization between three- and five-membered metallacycles was similarly reported by Diaconescu *et al.* for rare-earth metal complexes. Although the isomerization trend is opposite to the report by Diaconescu *et al.*, they mentioned that the pyridyl carbanion is more stable (2.8 kcal mol^–1^) than the phenyl carbanion for the phenylpyridyl anion. We presume that the relative stabilities of the three- and five-membered metallacycles are significantly affected by the attached metal fragment.^19^ The effect of substituents of the 2-arylpyridines on the C–C bond formation as shown in Scheme 4 indicates that the dissociation of the coordinating THF from the yttrium is key for further isomerization. Although the three-membered metallacycle intermediate **E** dimerized as a doubly μ,κ^2^-(C,N)-bridged dinuclear structure, similar to diyttrium complexes **6a–d**, introduction of aryl groups at the *ortho*-position of the pyridine ring might destabilize the μ,κ-(C,N)-bridging mode of the pyridine moiety to afford **4a** through 3-centered-2-electron aryl-bridged intermediate **F**.

## Conclusions

We demonstrated that yttrium-mediated 2,2′-bipyridyl formation proceeded through a bimetallic pyridylyttrium intermediate. Introduction of aryl substituents at the *ortho*-position of the pyridine ring destabilized the μ,κ^2^-(C,N)-bridged intermediate to accelerate associative bimetallic C(sp^2^)–C(sp^2^) bond formation. Further application of such bimetallic-mediated coupling reactions with not only rare-earth metal complexes but also early transition metal complexes is ongoing in our laboratory.
