# Supplementary material for: 2,2′-Bipyridyl formation from 2-arylpyridines through bimetallic diyttrium intermediate
Source: Chem Sci. 2015 Jul 17;6(10):5394–9. doi: 10.1039/c5sc01599e (PMC5510530; doi:10.1039/c5sc01599e)
Supplement: Supplementary file 1 [file SC-006-C5SC01599E-s001.pdf]

Electronic Supplementary Material (ESI) for Chemical Science.
This journal is © The Royal Society of Chemistry 2015

Supporting Information for

2,2'-Bipyridyl Formation from 2-Arylpyridines through Bimetallic Diyttrium Intermediate

Yu Shibata, Haruki Nagae, Shiki Sumiya, Raphaël Rochat, Hayato Tsurugi,*
and Kazushi Mashima*

Department of Chemistry, Graduate School of Engineering Science, Osaka University, and CREST, JST, Toyonaka, Osaka 560-8531, Japan

General: All manipulations involving air- and moisture-sensitive organometallic compounds were performed under argon using the standard Schlenk technique or an argon-filled glovebox. $ArNHCH_2CH_2NHAr$ (Ar = 2,6-$^{i}Pr_2C_6H_3$),[S1] $Y(CH_2SiMe_3)_3(THF)_2$,[S2] $Y(ArNCH_2CH_2NAr)(CH_2SiMe_3)_3(THF)_2$ (**1**),[S3] 2-(4-methylphenyl)pyridine (**2b**), and 2-phenylpyridine-d_5[S4] were prepared according to the literature procedure. Other pyridine derivatives were purchased and distilled over CaH_2. Anhydrous pentane, hexane, toluene, and THF were purchased from Kanto Chemical and further purified by passage through activated alumina columns under positive argon pressure as described by Grubbs *et al.*[S5] Benzene, $(Me_3Si)_2O$, benzene-d_6, toluene-d_8, and THF-d_8 were dried over CaH_2 and stored in glove box. ^{1}H NMR (400 MHz) and ^{13}C NMR (100 MHz) spectra were measured on a BRUKER AVANCEIII-400 spectrometer. Assignment of ^{1}H and $^{13}C\{^{1}H\}$ NMR peaks for some of the complexes was facilitated by 2D ^{1}H-^{1}H COSY, 2D ^{1}H-^{1}H NOESY, 2D ^{1}H-^{13}C HMQC, and 2D ^{1}H-^{13}C HMBC spectra. Elemental analyses were performed on a Perkin-Elmer 2400 microanalyzer at the Faculty of Engineering Science, Osaka University.

Preparation of Diyttrium Complex Bridged by 6,6'-diphenyl-2,2'-bipyridyl (4a):

A solution of 2-phenylpyridine (**2a**, 11.0 mg, 0.072 mmol) in benzene (0.5 mL) was added to a solution of **1** (50.0 mg, 0.072 mmol) in benzene (0.5 mL) by pipet. The color of the solution turned to green with green-colored powders. The solution was standing for 26 days, and large green crystals were precipitated. After decantation and washing with benzene (1 mL x 2) and pentane (1 mL), dark-green powders of **4a** were obtained quantitatively (51.6 mg, 0.0364 mmol). mp: 116–120 °C (dec.). The dark-green powders were insoluble in non-polar solvents such as C_6D_6 and $C_6D_5CD_3$. Anal Calcd for $(C_{82}H_{106}N_6O_2Y_2)\cdot(C_6H_6)$: C, 72.21; H, 7.71; N, 5.74. Found: C, 72.56; H, 7.82; N, 5.50.

Preparation of 4b:

Complex **4b** was obtained quantitatively (52 mg, 0.036 mmol), following similar procedure to the synthesis of **4a**: mp: 116–120 °C (dec.). Anal Calcd for $(C_{84}H_{110}N_6O_2Y_2)\cdot(C_6H_6)$: C, 72.46; H, 7.84; N, 5.63. Found: C, 72.75; H, 7.79; N, 5.32.

Preparation of 4c:

A solution of 2-(4-methoxyphenyl)pyridine (**2c**, 13.0 mg, 0.072 mmol) in benzene (0.5 mL) was added to a solution of **1** (50.0 mg, 0.072 mmol) in benzene (0.5 mL) by pipet in an Ar-filled glovebox. The reaction mixture was heated to 50 °C for 68 h to become green suspension. After decantation and washing with benzene (1 mL x 4) and pentane (1 mL), dark-green powders of **4c** were obtained in 36% yield (18.8 mg, 1.29 x 10^{-2} mmol). mp: 118–125 °C (dec.). Anal. Calcd. for $(C_{82}H_{106}N_6O_2Y_2)\cdot(C_6H_6)_2$: C, 71.98; H, 7.68; N, 5.25. Found: C, 71.60; H, 7.70; N, 5.49.

Preparation of ($ArNCH_2CH_2NAr$)Y(2-pyridylphenyl)$(THF)_2$ (3a):

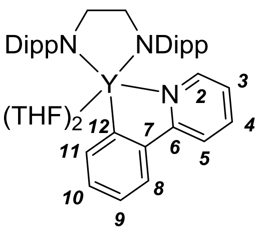

A solution of 2-phenylpyridine (**2a**, 22.3 mg, 0.144 mmol) in THF (1 mL) was added to a solution of complex **1** (100 mg, 0.144 mmol) in THF (1 mL) at room temperature by pipet in an Ar-filled glovebox, and the reaction mixture was stirred for 216 h. All of the solvent evaporated under reduced pressure, and the residue was washed with $(Me_3Si)_2O$ (1 mL x 5). By drying the formed precipitates in vacuo, yellow powders of **3a** were obtained in 98% yield (107 mg, 0.141 mmol). mp: 72–76 °C (dec.). ^{1}H NMR (400 MHz, THF-d_8, 30 °C) δ 8.26 (d, $^3J_{H\text{-}H}$ = 6.5 Hz, 1H, H^{11} of *Ph*Py), 8.20 (d, $^3J_{H\text{-}H}$ = 4.6 Hz, 1H, H^2 of Ph*Py*), 8.02 (d, $^3J_{H\text{-}H}$ = 8.3 Hz, 1H, H^8 of *Ph*Py), 7.77 (d, $^3J_{H\text{-}H}$ = 7.7 Hz, 1H, H^5 of Ph*Py*), 7.74 (t, $^3J_{H\text{-}H}$ = 7.7 Hz, 1H, H^4 of Ph*Py*), 7.69–7.12 (m, 3H, H^{10}, H^9, and H^3 of PhPy), 6.83 (d, $^3J_{H\text{-}H}$ = 7.5 Hz, 4H, *m*-H of 2,6-^{i}Pr$_2$C$_6$*H*$_3$), 6.65 (t, $^3J_{H\text{-}H}$ = 7.5 Hz, 2H, *p*-H of 2,6-^{i}Pr$_2$C$_6$*H*$_3$), 4.09 (sept, $^3J_{H\text{-}H}$ = 6.8 Hz, 4H, C*H*(CH$_3$)$_2$), 3.78 (s, 4H, N-CH$_2$), 3.61 (m, 8H, THF), 1.77 (q, $^3J_{H\text{-}H}$ = 3.2 Hz, 8H, THF), 1.04 (d, $^3J_{H\text{-}H}$ = 6.8 Hz, 24H, CH(C*H*$_3$)$_2$). ^{13}C NMR (100 MHz, THF-d_8, 30 °C) δ 190.3 (d, $^1J_{Y\text{-}C}$ = 48.8 Hz, Y-C), 165.0 (C^6 of Ph*Py*), 156.5 (*ipso*-C of 2,6-^{i}Pr$_2$C$_6$H$_3$), 146.8 (C^2 of Ph*Py*), 144.4 (C^7 of *Ph*Py), 143.7 (*o*-C of 2,6-^{i}Pr$_2$C$_6$H$_3$), 137.8 (C^{11} of *Ph*Py), 137.8 (C^4 of Ph*Py*), 125.8 (C^{10} of *Ph*Py), 123.4 (C^9 of *Ph*Py), 122.2 (C^8 of *Ph*Py), 121.2 (*m*-C of 2,6-^{i}Pr$_2$*C*$_6$H$_3$), 119.4 (C^3 of Ph*Py*), 118.9 (*p*-C of 2,6-^{i}Pr$_2$C$_6$*H*$_3$), 118.7 ($C^5$of Ph*Py*), 66.3 (THF), 59.6 (N-CH$_2$), 26.5 (*C*H(CH$_3$)$_2$), 24.4 (CH(*C*H$_3$)$_2$), 23.4 (THF). Anal. Calcd. for $C_{45}H_{60}N_3O_2Y$: C, 70.75; H, 7.92; N, 5.50. Found: C, 70.38; H, 8.17; N, 5.68.

Preparation of ($ArNCH_2CH_2NAr$)Y(2-pyridyl-4-MeOC$_6$H$_3$)$(THF)_2$ (3c):

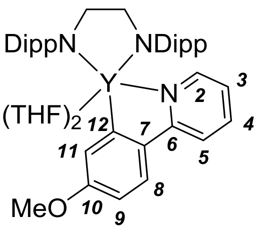

Complex **1** (50.2 mg, 0.0720 mmol) and 2-(4-methoxyphenyl)pyridine (**2c**, 13.3 mg, 0.0718 mmol) were dissolved in benzene (0.5 mL) in an Ar-filled glovebox. The reaction mixture was stirred at room

temperature for 3 h. After evaporating the solvent and washing with pentane (1 mL x 2), yellow powders of **3c** were obtained in 83% yield (47.7 mg, 0.0601 mmol). mp: 91–96 °C (dec.). ^{1}H NMR (400 MHz, C_6D_6, 30 °C) δ 8.09 (brs, 1H, H^2 of Ar*py*), 7.79-7.65 (m, 2H, H^{11} and H^8 of *Ar*py), 7.49 (d, $^3J_{H\text{-}H}$ = 8.3 Hz, 1H, H^5 of Ar*py*), 7.22 (d, $^3J_{H\text{-}H}$ = 7.5 Hz, 4H, *m*-H of 2,6-^{i}Pr$_2$C$_6$*H*$_3$), 7.10 (d, $^3J_{H\text{-}H}$ = 7.5 Hz, 2H, *p*-H of 2,6-^{i}Pr$_2$C$_6$H$_3$), 7.01 (m, 1H, H^4 of Ar*py*), 6.82 (d, $^3J_{H\text{-}H}$ = 8.3 Hz, 1H, H^9 of *Ar*py), 6.39 (t, $^3J_{H\text{-}H}$ = 6.2 Hz, 1H, H^3 of Ar*py*), 4.26-4.10 (m, 8H, N-CH_2 and C*H*(CH_3)$_2$), 3.61 (s, 3H, OCH_3), 3.40 (brs, 8H, THF), 1.30 (d, $^3J_{H\text{-}H}$ = 6.7 Hz, 24H, CH(C*H*$_3$)$_2$), 1.09 (brs, 8H, THF). ^{13}C NMR (100 MHz, C_6D_6, 30 °C) δ 194.1 (d, $^1J_{Y\text{-}C}$ = 46.8 Hz, Y-C^{12}), 166.0 (d, $^2J_{Y\text{-}C}$ = 2.4 Hz, C^6 of Ar*Py*), 159.7 (d, $^2J_{Y\text{-}C}$ = 1.3 Hz, C^7 of *Ar*py), 156.8 (*ipso*-C of 2,6-^{i}Pr$_2$C$_6$H$_3$), 147.7 (C^2 of Ar*py*), 145.1 (*o*-C of 2,6-^{i}Pr$_2$*C*$_6$H$_3$), 138.6 (C^4 of Ar*py*), 133.9 (C^{10} of *Ar*py), 125.3 (C^8 of *Ar*py), 123.3 (*m*-C of 2,6-^{i}Pr$_2$C$_6$H$_3$), 123.2 (C^{11} of *Ar*py), 121.3 (*p*-C of 2,6-^{i}Pr$_2$C$_6$H$_3$), 119.2 (C^3 and C^5 of Ar*py*), 111.8 (C^9 of *Ar*py), 69.8 (THF), 60.7 (N-CH_2), 54.6 (OCH_3), 28.3 (*C*H(CH_3)$_2$), 25.6 (CH(*C*H$_3$)$_2$), 25.0 (THF). Anal. Calcd for $C_{46}H_{62}N_3O_3Y_2$: C, 69.59; H, 7.87; N, 5.29. Found: C, 69.30; H, 7.96; N, 5.63.

Preparation of (ArNCH$_2$CH$_2$NAr)Y(2-pyridyl-4-CF$_3$C$_6$H$_3$)(THF)$_2$ (3d):

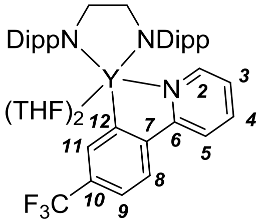

Complex **3d** was obtained in 83% yield (49.5 mg, 0.0595 mmol), following similar procedure to the synthesis of **3c**. mp: 94-99 °C (dec.). ^{1}H NMR (400 MHz, C_6D_6, 30 °C) δ 8.56 (s, 1H, H^{11} of *Ar*py), 8.33 (d, $^3J_{H\text{-}H}$ = 4.8 Hz, 1H, H^2 of Ar*py*), 7.51 (d, $^3J_{H\text{-}H}$ = 8.3 Hz, 1H, H^8 of *Ar*py), 7.50 (d, $^3J_{H\text{-}H}$ = 8.3 Hz, 1H, H^9 of *Ar*py), 7.44 (d, $^3J_{H\text{-}H}$ = 8.2 Hz, 1H, H^5 of Ar*py*), 7.18 (d, $^3J_{H\text{-}H}$ = 7.5 Hz, 4H, *m*-H of 2,6-^{i}Pr$_2$C$_6$*H*$_3$), 7.11-7.02 (m, 3H, *p*-H of 2,6-^{i}Pr$_2$C$_6$*H*$_3$ and H^4 of Ar*py*), 6.49 (t, $^3J_{H\text{-}H}$ = 6.3 Hz, 1H, H^3 of Ar*py*), 4.13 (m, 8H, N-CH_2 and C*H*(CH_3)$_2$), 3.37 (t, $^3J_{H\text{-}H}$ = 6.1 Hz, 8H, THF), 1.27 (d, $^3J_{H\text{-}H}$ = 6.8 Hz, 24 H, CH(C*H*$_3$)$_2$), 1.08 (m, 8H, THF). ^{13}C NMR (100 MHz, C_6D_6, 30 °C) δ 191.7 (d, $^1J_{Y\text{-}C}$ = 45.3 Hz, Y-C^{12}), 164.5 (d, $^2J_{Y\text{-}C}$

= 2.4 Hz, C^6 of Ar*py*), 156.6 (d, $^2J_{Y\text{-}C}$ = 2.9 Hz, *ipso*-C of 2,6-^{i}Pr$_2$C$_6$H$_3$), 149.2 (C^7 of *Ar*py), 148.4 (C^2 of Ar*py*), 144.9 (*o*-C of 2,6-^{i}Pr$_2$C$_6$H$_3$), 139.1 (C^4 of Ar*py*), 135.2 (d, $^3J_{C\text{-}F}$ = 3.3 Hz, C^2 of Ar*py*), 126.5 (q, $^1J_{C\text{-}F}$ = 273.0 Hz, CF$_3$), 123.3 (C^8 of *Ar*py), 123.3 (*m*-C of 2,6-^{i}Pr$_2$*C$_6$*H$_3$), 122.3 (d, $^2J_{C\text{-}F}$ = 3.9 Hz, C^9 of Ar*py*), 121.5 (*p*-C of 2,6-^{i}Pr$_2$*C$_6$*H$_3$), 121.2 (C^3 of Ar*py*), 120.2 (C^5 of Ar*py*), 70.0 (THF), 60.6 (N-CH$_2$), 28.3 (*C*H(CH$_3$)$_2$), 25.5 (CH(*C*H$_3$)$_2$), 24.9 (THF). ^{13}C NMR signal of *ipso*-C of CF$_3$ is not able to determine because their signal is overlapped with the signal of *C*$_6$D$_5$H. Anal. Calcd for C$_{46}$H$_{59}$F$_3$N$_3$O$_2$Y: C, 66.41; H, 7.15; N, 5.05. Found: C, 66.45; H, 7.51; N, 5.34.

Preparation of (ArNCH$_2$CH$_2$NAr)Y(κ-(*C*^{10},*N*)-benzo[*h*]quinolinyl)(THF)$_2$ (3d):

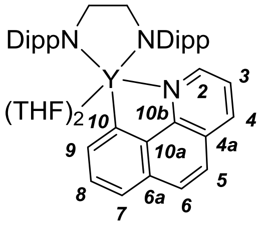

Complex **3d** was obtained quantitatively (59.5 mg, 0.0755 mmol), following similar procedure to the synthesis of **3c**. mp: 95-100 °C (dec.). ^{1}H NMR (400 MHz, C$_6$D$_6$, 30 °C) δ 8.56 (dd, $^3J_{H\text{-}H}$ = 4.9, 1.4 Hz, 1H, H^2), 8.51 (d, $^3J_{H\text{-}H}$ = 6.0 Hz, 1H, H^9), 7.71–7.58 (m, 3H, H^4, H^7, and H^8), 7.69 (d, $^3J_{H\text{-}H}$ = 8.8 Hz, 1H, H^6), 7.22 (d, $^3J_{H\text{-}H}$ = 8.8 Hz, 1H, H^5), 7.19 (d, $^3J_{H\text{-}H}$ = 7.8 Hz, 4H, *m*-H of 2,6-^{i}Pr$_2$C$_6$*H*$_3$), 7.08 (t, $^3J_{H\text{-}H}$ = 7.5 Hz, 2H, *o*-H of 2,6-^{i}Pr$_2$C$_6$*H*$_3$), 6.83 (dd, $^3J_{H\text{-}H}$ = 7.9, 4.9 Hz, 1H, H^3), 4.23 (s, 4H, N-CH$_2$), 4.22 (t, $^3J_{H\text{-}H}$ = 6.8 Hz, 4H, C*H*(CH$_3$)$_2$), 3.42 (s, 8H, THF), 1.20 (d, $^3J_{H\text{-}H}$ = 6.8 Hz, 24H, CH(C*H*$_3$)$_2$), 1.08 (s, 8H, THF). ^{13}C NMR (100 MHz, C$_6$D$_6$, 30 °C): δ 189.1 (d, $^1J_{Y\text{-}C}$ = 47.7 Hz, Y-C^{10}), 156.9 (*ipso*-C of 2,6-^{i}Pr$_2$C$_6$H$_3$), 154.8 (C^{10b}), 146.9 (C^2), 145.1 (*o*-C of 2,6-^{i}Pr$_2$C$_6$H$_3$), 138.2 (C^4), 137.9 (C^9), 133.6 (C^{6a}), 131.4 (C^6), 128.4 (C^{10a}), 127.8 (C^8), 127.3 (C^{4a}), 124.8 (C^7), 123.3 (*m*-H of 2,6-^{i}Pr$_2$C$_6$H$_3$), 122.3 (C^5), 121.3 (*p*-H of 2,6-^{i}Pr$_2$C$_6$H$_3$), 119.6 (C^3), 69.7 (THF), 60.8 (N-CH$_2$), 28.3 (*C*H(CH$_3$)$_2$), 25.5 (CH(*C*H$_3$)$_2$), 25.1(THF). Anal. Calcd for C$_{71}$H$_{60}$N$_3$O$_2$Y: C, 71.65; H, 7.68; N, 5.33. Found: C, 71.43; H, 7.82; N, 5.07.

Preparation of Bis(μ,κ–(C,N)-pyridyl)diyttrium Complex 6a:

A solution of pyridine (**5a**, 11.3 mg, 0.143 mmol) in benzene (1 mL) was added to a solution of **1** (100 mg, 0.144 mmol) in benzene (1 mL) by pipet in an Ar-filled glovebox. The reaction mixture was stand for 48 h at room temperature to form yellow precipitates. After decantation and washing with pentane (1 mL x 5), yellow powders of **6a** were obtained in 43% yield (38 mg, 0.031 mmol). mp: 79–82 °C (dec.). ^{1}H NMR (400 MHz, C_6D_6, 30 °C) δ 9.18 (d, $^{3}J_{H\text{-}H}$ = 7.4 Hz, 2H, 3-H of Py), 8.81 (d, $^{3}J_{H\text{-}H}$ = 5.0 Hz, 2H, 6-H of Py), 7.46 (t, $^{3}J_{H\text{-}H}$ = 7.4 Hz, 2H, 4-H of Py), 6.99–6.86 (m, 12H, 2,6-$^{i}Pr_2C_6H_3$), 6.80 (t, $^{3}J_{H\text{-}H}$ = 7.4 Hz, 2H, 5-H of Py), 4.12 (brs, 8H, N-CH_2), 3.94 (sept, $^{3}J_{H\text{-}H}$ = 6.8 Hz, 8H, C*H*(CH_3)$_2$), 2.88 (brs, 8H, α-THF), 1.13 (d, $^{3}J_{H\text{-}H}$ = 6.8 Hz, 48H, CH(C*H*$_3$)$_2$), 0.68 (brs, 8H, β-THF). Anal. Calcd. for $C_{70}H_{98}N_6O_2Y_2$: C, 68.17; H, 8.01; N, 6.81. Found: C, 68.12; H, 8.00; N, 7.16.

Complexes **6b-6d** were prepared according to the similar procedure to **6a**.

Complex **6b** was obtained in 38% yield (34.2 mg, 0.0271 mmol). mp: 82–86 °C (dec.). ^{1}H NMR (400 MHz, C_6D_6, 30 °C) δ 9.11 (s, 2H, 3-H of 4-Me*Py*), 8.73 (d, $^{3}J_{H\text{-}H}$ = 5.0 Hz, 2H, 6-H of 4-Me*Py*), 7.04 – 6.80 (m, 12H, 2,6-$^{i}Pr_2C_6H_3$), 6.75 (d, $^{3}J_{H\text{-}H}$ = 5.0 Hz, 2H, 5-H of 4-Me*Py*), 4.13 (brs, 8H, CH_2), 4.00 (sept, $^{3}J_{H\text{-}H}$ = 6.4 Hz, 8H, C*H*(CH_3)$_2$), 2.78 (brs, 8H, α-THF), 2.31 (s, 6H, 4-*Me*Py), 1.16 (br, 48H, CH(C*H*$_3$)$_2$), 0.61 (brs, 8H, β-THF). Anal. Calcd. for $C_{72}H_{102}N_6O_2Y_2$: C, 68.55; H, 8.15; N, 6.66. Found: C, 68.41; H, 8.49; N, 6.69.

Complex **6c** was obtained in 66% yield (58.8 mg, 0.0424 mmol). mp: 92–96 °C (dec.). ^{1}H NMR (400 MHz, C_6D_6, 30 °C) δ 9.68 (s, 2H, 3-H of Py), 8.90 (s br, 2H, 6-H of Py), 7.97 (d, ^{3}J = 6.6 Hz, 4H, *o*-H of *Ph*Py), 7.36 (t, ^{3}J = 7.3 Hz, 4H, *m*-H of *Ph*Py), 7.27-7.18 (m, 4H, 5-H of Py and *p*-H of *Ph*Py), 7.02-6.83 (m, 12H, 2,6-$^{i}Pr_2C_6H_3$), 4.60-3.80 (m, 16H, C*H*(CH_3)$_2$ and N-CH_2), 2.90 (brs, 8H, α-THF), 1.6-0.9 (br, 48H, CH(C*H*$_3$)$_2$), 0.63 (brs, 8H, β-THF). Anal. Calcd. for $C_{82}H_{106}N_6O_2Y_2$: C, 71.08; H, 7.71; N, 6.07. Found: C, 70.86; H, 7.42; N, 5.70.

Complex **6d** was obtained in 39% yield (36.3 mg, 0.0281 mmol). mp: 98–103 °C (dec.). ^{1}H NMR (400 MHz, Toluene-d_8, -60 °C) δ 8.95 (d, ^3J = 5.0 Hz, 2H, 6-H of Py), 6.99-6.94 (m, 12H, 2,6-^{i}Pr$_2$C$_6$$H_3$), 6.74 (m, 2H, 5-H of Py), 6.25 (d, ^3J = 8.4 Hz, 2H, 4-H of Py), 4.39-4.00 (m, 16H, N-CH$_2$ and CH(CH$_3$)$_2$), 3.88 (s, 6H, O-CH$_3$), 2.48 (brs, 8H, α-THF), 1.44 (br d, ^3J = 5.9 Hz, 12H, CH(CH_3)$_2$), 1.34 (brd, ^3J = 5.9 Hz, 12H, CH(CH_3)$_2$), 0.87 (brd, ^3J = 5.9 Hz, 12H, CH(CH_3)$_2$), 0.70 (brd, ^3J = 5.9 Hz, 12H, CH(CH_3)$_2$), 0.56-0.30 (m, 8H, β-THF). Anal. Calcd for $C_{72}H_{101}N_6O_4Y_2$: C, 66.91; H, 7.88; N, 6.50. Found: C, 67.16; H, 8.22; N, 6.80.

Preparation of κ–(C,N)-pyridylyttrium Complex 7e:

A solution of 3,5-lutidine (**5e**, 3.0 mg, 0.028 mmol) in C_6D_6 (0.5 mL) was added to **1** (20.0 mg, 0.0287 mmol) in C_6D_6 (0.5 mL) by pipet in an Ar-filled glovebox. The reaction mixture was heated to 60 °C for 24 h to quantitatively form **7e**. Isolation as powder form is difficult due to the high solubility of **7e** in any organic solvents. Complex **7e** was characterized by ^{1}H and ^{13}C NMR spectra. ^{1}H NMR (400 MHz, C_6D_6, 30 °C) δ 7.97 (s, 1H, 6-H of 3,5-Me$_2$*Py*), 7.21 (d, $^3J_{H\text{-}H}$ = 7.5 Hz, 4H, *m*-H of 2,6-^{i}Pr$_2$C$_6$H$_3$), 7.10 (t, $^3J_{H\text{-}H}$ = 7.5 Hz, 2H, *p*-H of 2,6-^{i}Pr$_2$C$_6$H$_3$), 6.88 (s, 1H, 4-H of 3,5-Me$_2$*Py*), 4.24 (sept, $^3J_{H\text{-}H}$ = 6.9 Hz, 4H, CH(CH$_3$)$_2$), 4.17 (brs, 4H, NCH$_2$), 3.48 (brs, 8H, α-THF), 2.65 (s, 3H, 3-Me of 3,5-*Me*$_2$Py), 1.91 (s, 3H, 5-Me of 3,5-*Me*$_2$Py), 1.37 (d, $^3J_{H\text{-}H}$ = 6.9 Hz, 24H, CH(CH_3)$_2$), 1.24 (brs, 8H, β-THF). ^{13}C NMR (100 MHz, C_6D_6, 30 °C) δ 219.6 (d, $^1J_{Y\text{-}C}$ = 35.2 Hz, Y-C), 157.1 (d, $^2J_{Y\text{-}C}$ = 2.8 Hz, *ipso*-C of 2,6-^{i}Pr$_2$C$_6$H$_3$), 145.0 (*o*-C of 2,6-^{i}Pr$_2$$C_6H_3$), 142.4 (6-C of 3,5-Me$_2$*Py*), 140.0 (d, $^2J_{Y\text{-}C}$ = 2.0 Hz, 3-C of 3,5-Me$_2$*Py*), 133.3 (d, $^3J_{Y\text{-}C}$ = 1.3 Hz, 4-C of 3,5-Me$_2$*Py*), 129.9 (5-C of 3,5-Me$_2$*Py*), 123.2 (*m*-C of 2,6-^{i}Pr$_2$$C_6H_3$), 120.9 (*p*-C of 2,6-^{i}Pr$_2$$C_6H_3$), 69.0 (α-THF), 61.0 (N-CH$_2$), 28.4 (CH(CH$_3$)$_2$), 25.7 (CH(CH$_3$)$_2$), 25.5 (β-THF), 23.8 (3-Me of 3,5-*Me*$_2$Py), 18.1 (5-Me of 3,5-*Me*$_2$Py). Due to the high solubility of the product, we could not isolate in powder form. The ^{1}H and ^{13}C NMR spectra were shown in Figure S4.

Preparation of $(ArNCH_2CH_2NAr)Y(CH_2SiMe_2Py)(THF)_2$ (8f):

A solution of pyridine (**5a**, 10.9 mg, 0.0721 mmol) in benzene (0.5 mL) was added to a solution of **1** (50.2 mg, 0.0720 mmol) in benzene (0.5 mL) by pipet in an Ar-filled glovebox. The reaction mixture was stand for 24 h at room temperature, and then all the solvent was evaporated. After washing with $(Me_3Si)_2O$ (1 mL x 5), drying the remaining solid in vacuo to give yellow powders of **8f** in 69% yield (37 mg, 0.049 mmol). mp: 111–113 °C (dec.). ^1H NMR (400 MHz, C_6D_6, 30 °C) δ 7.97 (d, $^3J_{H-H}$ = 5.2 Hz, 1H, 6-H of Py), 7.20-7.17 (m, 5H, *m*-H of 2,6-$^i Pr_2C_6H_3$ and 3-H of Py), 7.10 (t, $^3J_{H-H}$ = 7.5 Hz, 2H, *p*-H of 2,6-$^i Pr_2C_6H_3$), 6.83 (dt, $^3J_{H-H}$ = 7.6 Hz, $^4J_{H-H}$ = 1.4 Hz, 1H, 4-H of *Py*), 6.39-6.34 (m, 1H, 5-H of Py), 4.20 (sept, $^3J_{H-H}$ = 6.9 Hz, 4H, $CH(CH_3)_2$), 4.10 (s, 4H, NCH_2), 3.50 (brs, 8H, α-THF), 1.36 (d, $^3J_{H-H}$ = 6.9 Hz, 24H, $CH(CH_3)_2$), 1.17 (brs, 8H, β-THF), 0.29 (s, 6H, $SiMe_2$). ^{13}C NMR (100 MHz, C_6D_6, 30 °C) δ 179.0 (d, $^2J_{Y-C}$ = 1.8 Hz, 2-C of Py), 156.2 (d, $^2J_{Y-C}$ = 2.7 Hz, *ipso*-C of 2,6-$^i Pr_2C_6H_3$), 148.8 (6-C of Py), 145.5 (*o*-C of 2,6-$^i Pr_2C_6H_3$), 136.2 (4-C of Py), 130.6 (5-C of Py), 123.7 (*m*-C of 2,6-$^i Pr_2C_6H_3$), 122.3 (3-C of Py), 121.8 (*p*-C of 2,6-$^i Pr_2C_6H_3$), 69.7 (α-THF), 60.8 (N-CH_2), 28.7 ($CH(CH_3)_2$), 26.0 ($CH(CH_3)_2$), 25.5 (β-THF), 18.1 (d, $^1J_{Y-C}$ = 37.2 Hz, Y-C), 3.06 ($SiMe_2$). Anal. Calcd for $C_{42}H_{64}N_3O_2SiY$: C, 66.38; H, 8.49; N, 5.53. Found: C, 66.35; H, 8.54; N, 5.69.

Deuterium Labeling Experiment:

Diyttrium complex **3a-*d*₈,*d*₉,*d*₁₀** (100 mg, 0.0702 mmol) was suspended in C_6H_6 (2 mL), and CCl_4 (13.0 mg, 0.087 mmol, 1.2 equiv) was added to quench the reaction mixture. After heating the reaction mixture for 24 h at 60 °C, deuterated 6,6'-diphenyl-2,2'-bipyridyl was isolated by silica-gel column chromatography. ^1H NMR (400 MHz, C_6D_6, 30 °C) δ 8.61 (d, $^3J_{H-H}$ = 7.2 Hz, 2H, 3-H of Py), 8.18 (s, 1.4H, *o*-Ph), 7.92 (t, $^3J_{H-H}$ = 7.2 Hz, 2H, 4H of Py), 7.79 (d, $^3J_{H-H}$ = 7.2 Hz, 2H, 5-H of Py). The ^1H NMR of the isolated product was shown in Figure S5.

X-ray Crystallographic Studies

Single crystals of **3e**, **4a-c**, **6a**, **6c**, and **6d** were obtained from the corresponding saturated solution. Suitable crystals were mounted on a CryoLoop (Hampton Research Corp.) with a layer of light mineral oil and placed in a nitrogen stream at 113 K. Crystal data and structure refinement parameters were summarized in Table S3.

The structures of all these complexes were solved by a direct method (SIR92[S6] or SHELXS-97[S7]) and refined on F^2 by full-matrix least-squares methods, using SHELXL-97. Non-hydrogen atoms were anisotropically refined. H-atoms were included in the refinement on calculated positions riding on their carrier atoms. The function minimized was $[\Sigma w(Fo^2 - Fc^2)^2]$ ($w = 1 / [\sigma^2 (Fo^2) + (aP)^2 + bP]$), where $P = (\mathrm{Max}(Fo^2,0) + 2Fc^2) / 3$ with $\sigma^2(Fo^2)$ from counting statistics. The function $R1$ and $wR2$ were $(\Sigma||Fo| - |Fc||) / \Sigma|Fo|$ and $[\Sigma w(Fo^2 - Fc^2)^2 / \Sigma w(Fo^4)]^{1/2}$, respectively. The ORTEP-3 program[S8] was used to draw each molecule.

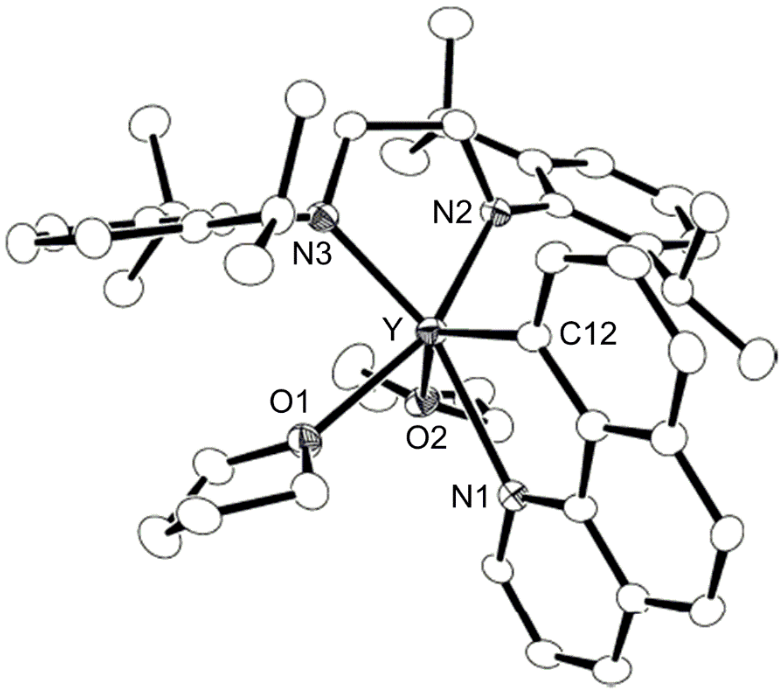

Figure S1. ORTEP drawing of complexes **3e** with 30% thermal ellipsoid. All hydrogen atoms and packing solvent molecules are omitted for clarity. Selected bond lengths (Å) and angles (°): Y – N1, 2.631(6); Y – N2, 2.272(6); Y – N3, 2.290(6); Y – C12, 2.497(7); Y – O1, 2.448(5); Y – O2, 2.443(5); N2 – Y – N3, 77.5(2); O1 – Y – O2, 84.95(18).

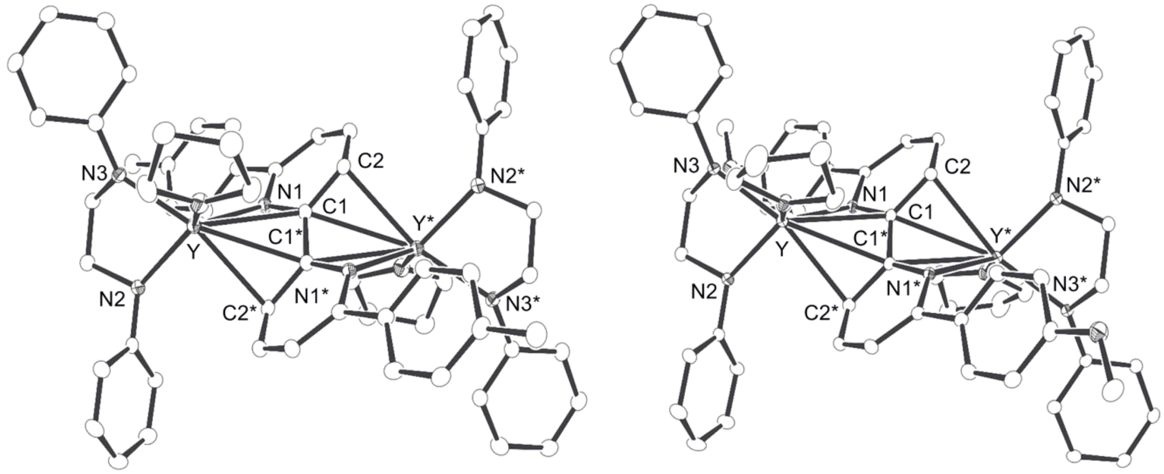

Figure S2. ORTEP drawing of complexes **4b** (left) and **4c** (right) with 30% thermal ellipsoid. All hydrogen atoms and packing solvent molecules are omitted for clarity.

Table S1. Selected geometries (bond lengths (Å) and angles (deg)) of complexes **4a-c**.

complex	**4a**	**4b**	**4c**
Y—N1	2.344(5)	2.335(3)	2.333(2)
Y—C1	2.746(5)	2.755(4)	2.771(2)
Y—C1*	2.857(5)	2.860(4)	2.888(2)
Y—C2*	2.661(5)	2.669(4)[a]	2.686(3)
N1—C1	1.424(6)	1.420(4)	1.429(3)
C1—C2	1.476(7)	1.465(5)	1.470(4)
C1—C1*	1.396(10)	1.418(7)	1.392(5)
Y—N2	2.194(4)	2.205(3)	2.213(2)
Y—N3	2.192(5)	2.200(3)	2.183(2)
N1—Y—C2*	70.68(16)	70.63(11)	70.14(8)
N2—Y—N3	80.78(17)	79.80(11)	80.60(8)
Dihedral angle between N1–Y–C2* and N1–C1–C1*–C2* planes	109.6	110.0	110.9

[a] Y—C2

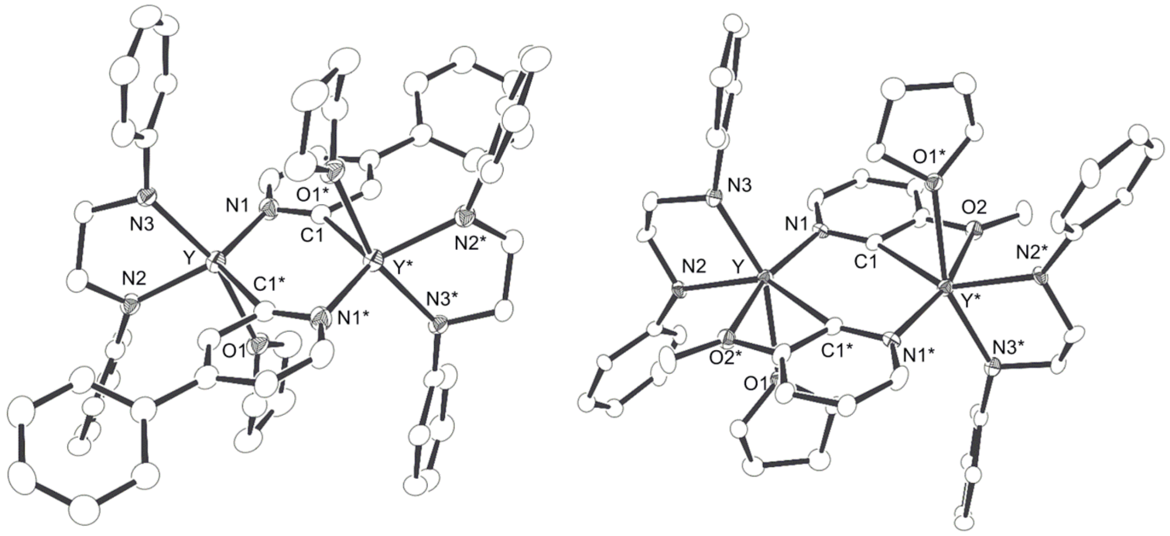

Figure S3. ORTEP drawing of complex **6c** (left) and **6d** (right) with 30% thermal ellipsoid. All hydrogen atoms and packing solvent molecules are omitted for clarity.

Table S2. Selected geometries (bond lengths (Å) and angles (deg)) of complexes **6a**, **6c**, and **6d**.

complex	**6a**	**6c**	**6d**
Y—N1	2.328(4)	2.352(5)	2.366(5)
Y—C1	2.681(5)	2.735(6)[a)]	2.850(7)[a)]
Y—C1*	2.560(5)	2.588(6)[b)]	2.685(7)[b)]
N1—C1	1.380(6)	1.379(7)	1.376(8)
Y—N2	2.216(4)	2.237(5)	2.239(6)
Y—N3	2.219(4)	2.210(5)	2.252(5)
Y—O1(THF)	2.422(3)	2.419(4)	-
Y—O1*	-	-	2.504(5)
Y—O2(THF)	-	-	2.404(5)

[a)] Y—C1* [b)] Y—C1

Table S3. Crystal Data and Data Collection Parameters for **3e**, **4a-c**, **6a**, **6c**, and **6d**.[a) b)]

compound	**3e**	**4a**
empirical formula	$C_{47}H_{62}N_3O_2Y$	$C_{82}H_{108}N_6O_2Y_2\cdot 2(C_6H_6)$
formula weight	789.93	1543.78
crystal system	*monoclinic*	*monoclinic*
space group	$P2_1/n$ (#14)	$P2_1/n$ (#14)
a, Å	12.902(11)	13.160(7)
b, Å	21.925(16)	16.367(8)
c, Å	16.088(13)	19.701(10)
α, deg.	-	-
β, deg.	106.222(7)	103.837(4)
γ, deg.	-	-
V, Å^3	4370(6)	4120(4)
Z	4	2
D_{calcd}, g/cm^3	1.201	1.244
μ [Mo-Kα], mm^{-1}	1.375	1.453
crystal size, mm^3	0.30×0.20×0.05	0.28×0.26×0.23
T, K	113(2)	113(2)
θ range for data collection, deg.	3.23 to 27.23	3.01 to 26.00
no. of reflections measured	36919	35619
unique data (R_{int})	8565 (0.1279)	8083 (0.1303)
data / restraints / parameters	8565 / 0 / 486	8083 / 12 / 471
$R1$ ($I > 2.0\sigma(I)$)[a)]	0.1188	0.0948
$wR2$ ($I > 2.0\sigma(I)$)[b)]	0.2648	0.1451
$R1$ (all data)[a)]	0.1641	0.1412
$wR2$ (all data)[b)]	0.3028	0.1664
GOF on F^2	1.111	1.127
$\Delta\rho$, e Å^{-3}	1.87, -0. 95	0.51, -0.495

a) $R1 = (\Sigma||Fo| - |Fc||)/(\Sigma|Fo|)$ b) $wR2 = [\{\Sigma w(Fo^2\text{-}Fc^2)^2\}/\{\Sigma w(Fo^4)\}]^{1/2}$

Table S3 (continued). Crystal Data and Data Collection Parameters for **3e**, **4a-c**, **6a**, **6c**, and **6d**.[a) b)]

compound	**4b**	**4c**
empirical formula	$C_{84}H_{110}N_6O_2Y_2\cdot 2(C_7H_8)$	$C_{84}H_{110}N_6O_4Y_2\cdot 2(C_7H_8)$
formula weight	1599.88	1631.94
crystal system	*monoclinic*	*monoclinic*
space group	$P2_1/n$ (#14)	$P2_1/n$ (#14)
a, Å	13.324(6)	13.2137(9)
b, Å	16.849(7)	17.8164(9)
c, Å	19.590(8)	18.8346(12)
α, deg.	-	-
β, deg.	104.350(5)	102.372(3)
γ, deg.	-	-
V, Å^3	4261(3)	4331.1(5)
Z	2	2
D_{calcd}, g/cm^3	1.247	1.251
μ [Mo-Kα], mm^{-1}	1.4095	1.3896
crystal size, mm^3	0.32×0.29×0.06	0.47×0.38×0.10
T, K	113(2)	113(2)
θ range for data collection, deg.	3.16 to 27.49	3.11 to 27.47
no. of reflections measured	40751	41389
unique data (R_{int})	9727 (0.0782)	9871 (0.0598)
data / restraints / parameters	9727 / 0 / 487	9871 / 0 / 496
$R1$ ($I > 2.0\sigma(I)$)[a)]	0.0661	0.0498
$wR2$ ($I > 2.0\sigma(I)$)[b)]	0.1386	0.1025
$R1$ (all data)[a)]	0.1024	0.0681
$wR2$ (all data)[b)]	0.1626	0.1130
GOF on F^2	1.061	1.093
$\Delta\rho$, e Å^{-3}	1.45, -0.75	0.78, -0.79

a) $R1 = (\Sigma||Fo| - |Fc||)/(\Sigma|Fo|)$ b) $wR2 = [\{\Sigma w(Fo^2\text{-}Fc^2)^2\}/\{\Sigma w(Fo^4)\}]^{1/2}$

Table S3 (continued). Crystal Data and Data Collection Parameters for **3e**, **4a-c**, **6a**, **6c**, and **6d**.[a) b)]

compound	**6a**	**6c**
empirical formula	$C_{70}H_{100}N_6O_2Y_2$	$C_{82}H_{106}N_6O_2Y_2 \cdot 2(C_6H_6)$
formula weight	1235.38	1543.83
crystal system	*monoclinic*	*monoclinic*
space group	*C*2/*c* (#15)	$P2_1/n$ (#14)
a, Å	16.313(7)	15.639(7)
b, Å	20.362(9)	17.514(8)
c, Å	20.231(9)	16.240(8)
α, deg.	-	-
β, deg.	92.019(4)	104.711(4)
γ, deg.	-	-
V, $Å^3$	6716(5)	4302(3)
Z	4	2
D_{calcd}, g/cm^3	1.222	1.192
μ [Mo-Kα], mm^{-1}	1.766	1.3935
crystal size, mm^3	0.20×0.08×0.08	0.21×0.17×0.10
T, K	113(2)	113(2)
θ range for data collection, deg.	3.15 to 26.00	3.13 to 27.47
no. of reflections measured	29121	35630
unique data (R_{int})	6581 (0.1065)	7992 (0.1016)
data / restraints / parameters	6581 / 0 / 369	7992 / 0 / 501
$R1$ ($I > 2.0\sigma(I)$)[a)]	0.0784	0.1176
$wR2$ ($I > 2.0\sigma(I)$)[b)]	0.1337	0.1838
$R1$ (all data)[a)]	0.1091	0.1348
$wR2$ (all data)[b)]	0.1492	0.1930
GOF on F^2	1.109	1.290
$\Delta\rho$, e $Å^{-3}$	1.214, -0.568	0.75, -0.69

a) $R1 = (\Sigma||Fo| - |Fc||)/(\Sigma|Fo|)$ b) $wR2 = [\{\Sigma w(Fo^2 - Fc^2)^2\}/\{\Sigma w(Fo^4)\}]^{1/2}$

Table S3 (continued). Crystal Data and Data Collection Parameters for **3e**, **4a-c**, **6a**, **6c**, and **6d**.[a) b)]

compound	**6d**
empirical formula	$C_{72}H_{104}N_6O_4Y_2$
formula weight	1295.46
crystal system	*monoclinic*
space group	$P2_1/n$ (#14)
a, Å	13.492(12)
b, Å	13.673(12)
c, Å	19.029(18)
α, deg.	-
β, deg.	101.334(9)
γ, deg.	-
V, Å^3	3442(5)
Z	2
D_{calcd}, g/cm^3	1.250
μ [Mo-Kα], mm^{-1}	1.7304
crystal size, mm^3	0.17×0.15×0.05
T, K	113(2)
θ range for data collection, deg.	3.17 to 27.48
no. of reflections measured	27295
unique data (R_{int})	6063 (0.1436)
data / restraints / parameters	6060 / 0 / 379
$R1$ ($I > 2.0\sigma(I)$)[a)]	0.0914
$wR2$ ($I > 2.0\sigma(I)$)[b)]	0.1563
$R1$ (all data)[a)]	0.1414
$wR2$ (all data)[b)]	0.1837
GOF on F^2	1.063
$\Delta\rho$, e Å^{-3}	1.51, -0.64

a) $R1 = (\Sigma||Fo| - |Fc||)/(\Sigma|Fo|)$ b) $wR2 = [\{\Sigma w(Fo^2-Fc^2)^2\}/\{\Sigma w(Fo^4)\}]^{1/2}$

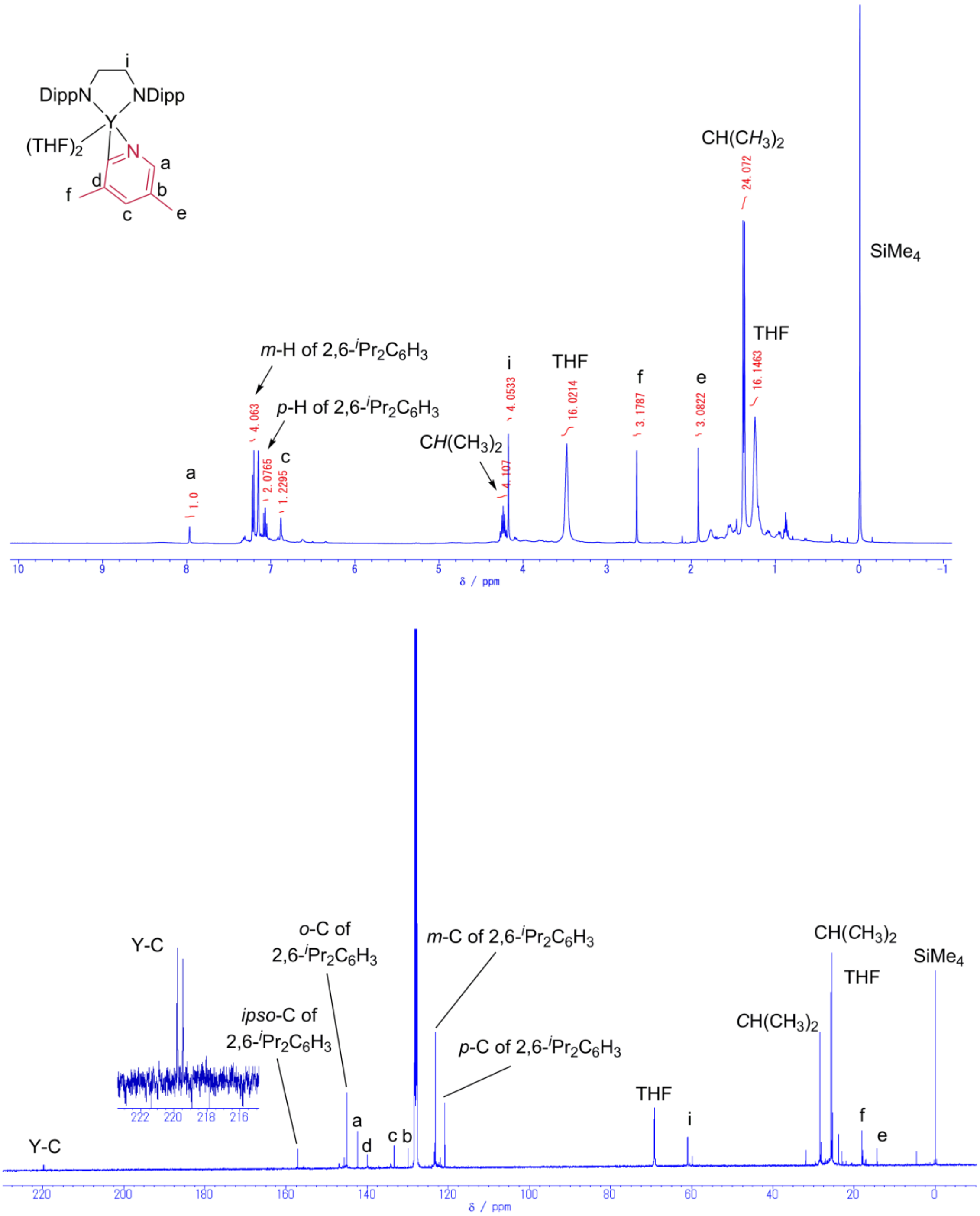

Figure S4. ^{1}H NMR (top) and ^{13}C NMR (bottomo) spectra of **7e**.

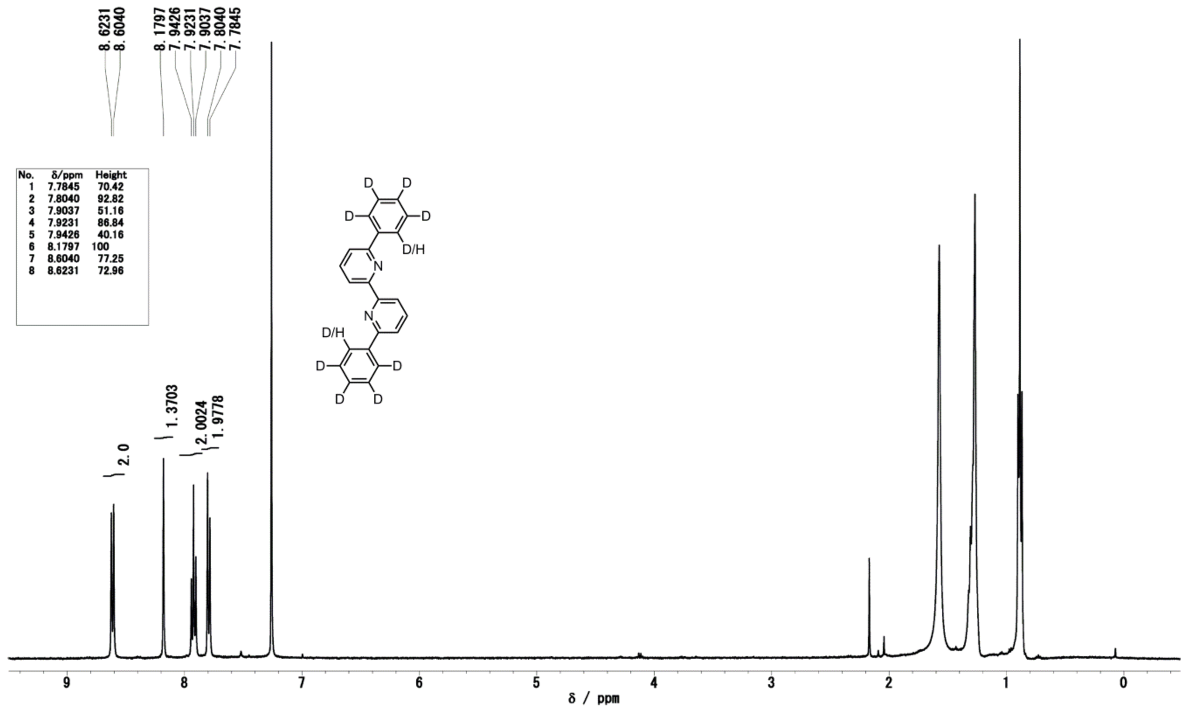

Figure S5. ^{1}H NMR spectrum of deuterated 6,6'-diphenyl-2,2'-bipyridyl.

References

S1) Abrams, M. B.; Scott, B. L.; Baker, R. T. *Organometallics* **2000**, *19*, 4944.

S2) Mazzeo, M.; Lamberti, M.; D'Auria, I.; Milione, S.; Peters, J. C.; Pellecchia, C. *J. Polym. Sci., Part A: Polym. Chem.* **2010**, *48*, 1374.

S3) Eppinger, J.; Nikolaides, K. R.; Zhang-Presse, M.; Riederer, F. A.; Rabe, G. W.; Rheingold, A. L. *Organometallics* **2008**, *27*, 736.

S4) Kozhushkov, S. I.; Yufit, D. S.; Ackermann, L. *Org. Lett.* **2008**, *10*, 3409.

S5) Pangborn, A. B.; Giardello, M. A.; Grubbs, R. H.; Rosen, R. K.; Timmers, F. J. *Organometallics* **1996**, *15*, 1518.

S6) Altomare, A.; Burla, M. C.; Camalli, M.; Cascarano, G. L.; Giacovazzo, C.; Guagliardi, A.; Polidori, G. *J. Appl. Cryst.* **1994**, *27*, 435.

S7) Sheldrich, G. M. *Acta Cryst.* **2008**, *A64*, 112.

S8) Farrugia, L. J. *J. Appl. Cryst.* **1999**, *32*, 837.
